# Temperature response of soil carbon decomposition depends strongly on forest management practice and soil layer on the eastern Tibetan Plateau

**DOI:** 10.1038/s41598-017-05141-2

**Published:** 2017-07-06

**Authors:** Kaijun Yang, Ruoyang He, Wanqin Yang, Zhijie Li, Liyan Zhuang, Fuzhong Wu, Bo Tan, Yang Liu, Li Zhang, Lihua Tu, Zhenfeng Xu

**Affiliations:** Long-term Research Station of Alpine Forest Ecosystems, Key Laboratory of Ecological Forestry Engineering, Institute of Ecology and Forest, Sichuan Agricultural University, Chengdu, 611130 China

## Abstract

How forest management practice impacts the temperature response of soil carbon decomposition remains unclear in Tibetan boreal forests. Here, an experiment was conducted to compare soil carbon decomposition of two layers (organic and mineral) in three Tibetan forests (natural forest, NF; secondary forest, SF; spruce plantation, PF). Soils were incubated at two temperatures (10 °C and 20 °C) for 219 days. Increased temperature often stimulated carbon decomposition rates of organic layer but did not affect them in the mineral soils. Soil carbon decomposition rates in the organic layer followed a pattern of NF > SF > PF over the incubation period. Regardless of forest type, soil carbon decomposition rates and temperature coefficient (*Q*_10_) were higher in the organic layers compared to mineral soils. Moreover, forest type conversion increased *Q*_10_ values in each soil layer. Taken together, our results suggest that forest management practice has much stronger impacts on biochemical properties in the organic layers relative to mineral soils. Moreover, the temperature responses of soil carbon decomposition depend largely on forest management practice and soil layer in this specific area.

## Introduction

Boreal forests cover one third of the world’s forested area and store about 30% of the global terrestrial carbon pool^1^. It is believed that boreal forest soil, acts as a key carbon pool, plays an increasingly important role in carbon (C) cycling of terrestrial ecosystems. Climate warming is predicted to affect almost all terrestrial ecosystems and will be particularly pronounced in cold biomes^2^. Temperature is considered to be a key factor that regulates the decomposition of soil organic matter (SOM), which is a large component of the terrestrial carbon budget^3, 4^. Thus, warming-associated increases in SOM decomposition could profoundly affect the carbon balance in boreal forest soils, with consequent feedbacks to global warming.

The temperature response of soil C decomposition may depend largely on the initial conditions of substrates, such as stocks of SOM, the chemical composition and microbial community^5–7^. In addition, soil responses to climate change could also be complicated by land-use change^8^. Forest management practice (e.g., artificial reforestation and natural regeneration) often produces significant changes in soil biochemical conditions, which in turn could directly and/or indirectly affect the response of soil C decomposition to climate change^9, 10^. As a consequence, it is very crucial to synchronously compare the temperature effects on soil C decomposition under different forest managements.

As well known, boreal forests accumulate a large amount of organic material in the surface forest floor (a thick organic layer) as a result of slow decomposition process. An organic layer often includes various stages of decomposed organic matter, such as highly decomposed, septic; moderately decomposed, hemic, and minimally decomposed^11^. Compared to mineral horizons in the soil profile, they are rich in organic matter, with typically black or dark brown in color. In boreal forest ecosystems, organic layer is considered to be the most active interface where many biochemical cycles between trees and soils occur^12^. The organic layer and the mineral soil often have different substrate quality and availability for the decomposition of SOM due to different rates of C input, accumulation, and turnover in both layers^13, 14^. Therefore, the temperature sensitivity of soil C decomposition could be differential between the two soil layers.

The Tibetan forests are typical alpine boreal forests at low latitude, with important consequences for regional and global carbon balance. The magnitude of climate change on the Tibetan Plateau is projected to be large relative to many other regions^15^. Additionally, a large amount of SOM is stored in the organic layer besides the mineral soil in Tibetan forests^16^. Therefore, soil C decomposition of Tibetan forests is likely to be more pronounced relative to other forest ecosystems in a warmer world. Over the last decades, the natural coniferous forests have been harvested in large-scale industrial logging, and replaced by secondary forests and dragon spruce plantation under national restoration programs^17^. Forest management practice (e.g., artificial reforestation or natural regeneration) often induces significant changes in soil physical and biochemical properties^12^, especially in the organic layer, which in turn might largely regulate the temperature responses of soil C decomposition. Here, an experiment was conducted to explore temperature effects on soil C mineralization of two layers (organic and mineral) in three contrasting forest ecosystems (natural forest, secondary forest and spruce plantation) on the eastern Tibetan Plateau. Specifically, we hypothesized that (1) forest land-use change would lower soil substrate quality and C decomposition; (2) temperature response of soil C decomposition would vary with forest types and soil layers.

## Results

### Soil properties

**Figure 1:**
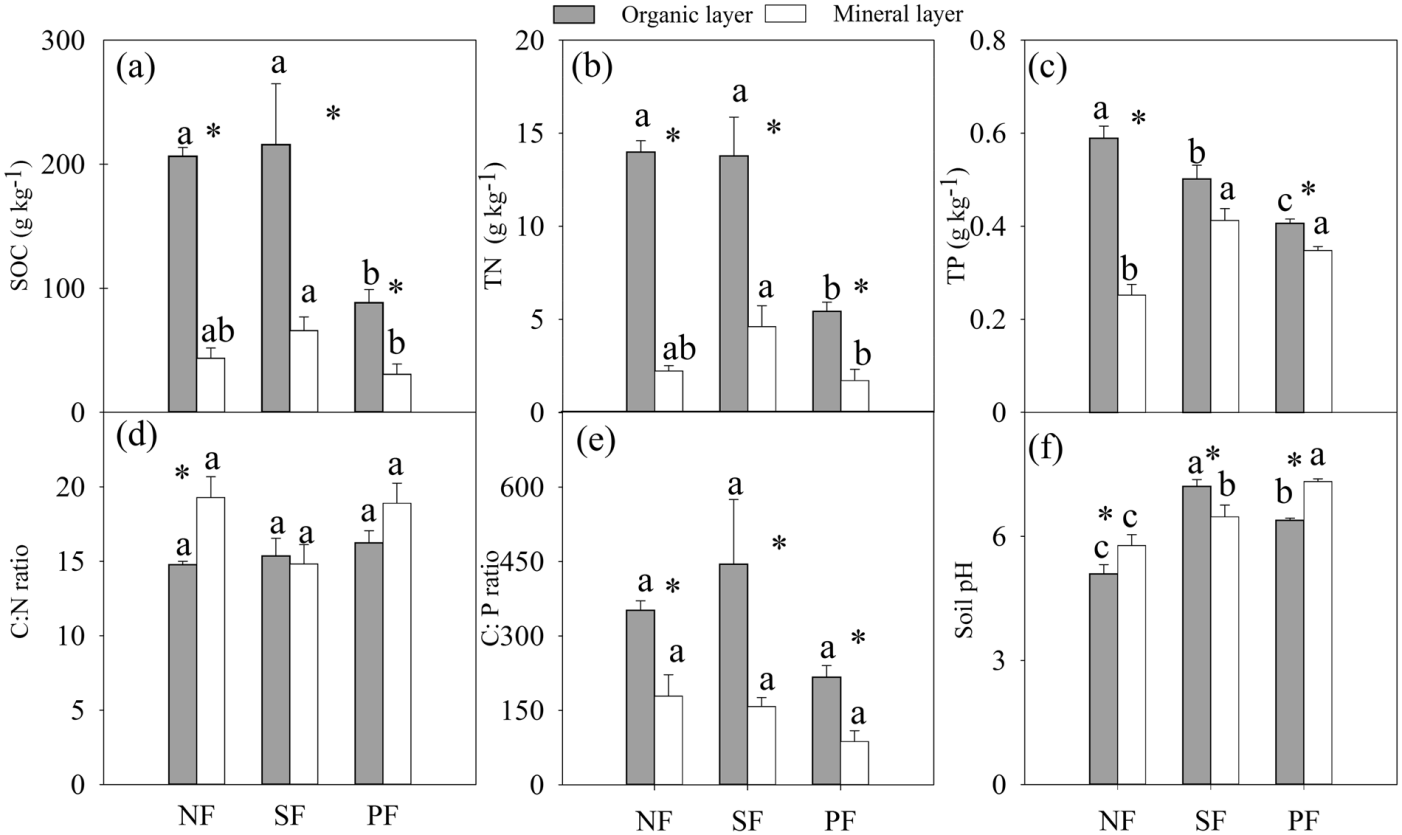
Effects of forest type conversion and soil layer on soil properties. Values indicate means ± SE, n = 4. Different letters within the same soil layer denote significant differences among forest types by one-way ANOVA. Asterisk indicates significant differences between the two soil layers by student *t*-test. NF: natural forest, SF: secondary forest and PF: spruce plantation.

Compared to the mineral soils, SOC, N and P in the organic layer were 2.9–4.7, 2.0–6.3 and 1.2–2.4 times higher among three forests (Fig. 1a,b and c, all *p* < 0.05). In the organic layer, SOC, N and P in both NF and SF were significantly higher than those in the PF (*p* < 0.05). However, SOC, N and P was greatest in the SF in the mineral soil (*p* < 0.05). Compared to the mineral soils, lower C:N ratio of the organic layer was only observed in the NF (Fig. 1d). Similarly, there were no significant differences in C:P ratio among the forests (Fig. 1e). However, C:P ratio was higher in the organic layer in each forest type as compared to mineral soil (*p* < 0.05). Soil pH increased from NF to SF or PF in both soil layers (Fig. 1f). The statistical analysis showed that the effect of forest conversion on SOC, N, P and pH were dependent on soil layer (Table 1).

**Table 1:** Results of two factors ANOVA showing the *F* and *P* values for responses of soil properties to forest type (FT) and soil layers (SL). *p < 0.05, **p < 0.01, ns: not significant.

Sources	SOC	N	P	pH	C:N	C:P	Bacteria	Fungi	Bacteria:Fungi	*Q*_10_
FT	7.85*	16.01**	7.98**	34.47**	2.69 ns	3.54 ns	6.14*	10.59**	6.24*	6.14**
SL	48.44**	90.75**	89.09**	3.34 ns	5.82*	16.95**	0.82**	49.08**	13.41*	6.18*
FT × SL	3.48*	7.56**	26.76**	10.49**	2.57 ns	0.96 ns	24.49**	7.13**	11.06**	0.42 ns

### Soil microbial community

**Figure 2:**
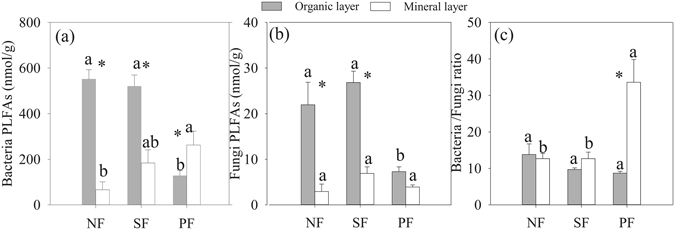
Effects of forest type conversion and soil layer on soil microbial properties. Values indicate means ± SE, n = 4. Different letters within the same soil layer denote significant differences among forest types by one-way ANOVA. Asterisk indicates significant differences between the two soil layers by student *t*-test. NF: natural forest, SF: secondary forest and PF: spruce plantation.

Bacteria, fungi and their ratio were significantly affected by forest conversion and soil layer (Fig. 2, Table 1). In the organic layer, bacteria and fungi PLFAs were markedly larger in both NF and SF compared to PF (Fig. 2a). In the mineral layer, bacteria PLFAs were 3.9 and 2.8 times greater, respectively, in the SF and PF compared to NF (Fig. 2a). Bacteria PLFAs in the organic layer were significantly higher as compared to mineral soil in both NF and SF. However, the opposite pattern was true in the PF (Fig. 2a). In addition, fungi PLFAs were higher in the organic layer than in the mineral soil except for the PF (Fig. 2b). However, forest conversion did not affect bacteria:fungi ratio in the organic layer (Fig. 2c). Additionally, obvious difference in bacteria:fungi ratio between soil layers was only observed in the PF (Fig. 2c). The ANOVA results showed that there were interactive effects of forest type and soil layer on bacteria, fungi and their ratio (Table 1).

### Soil C mineralization

**Figure 3:**
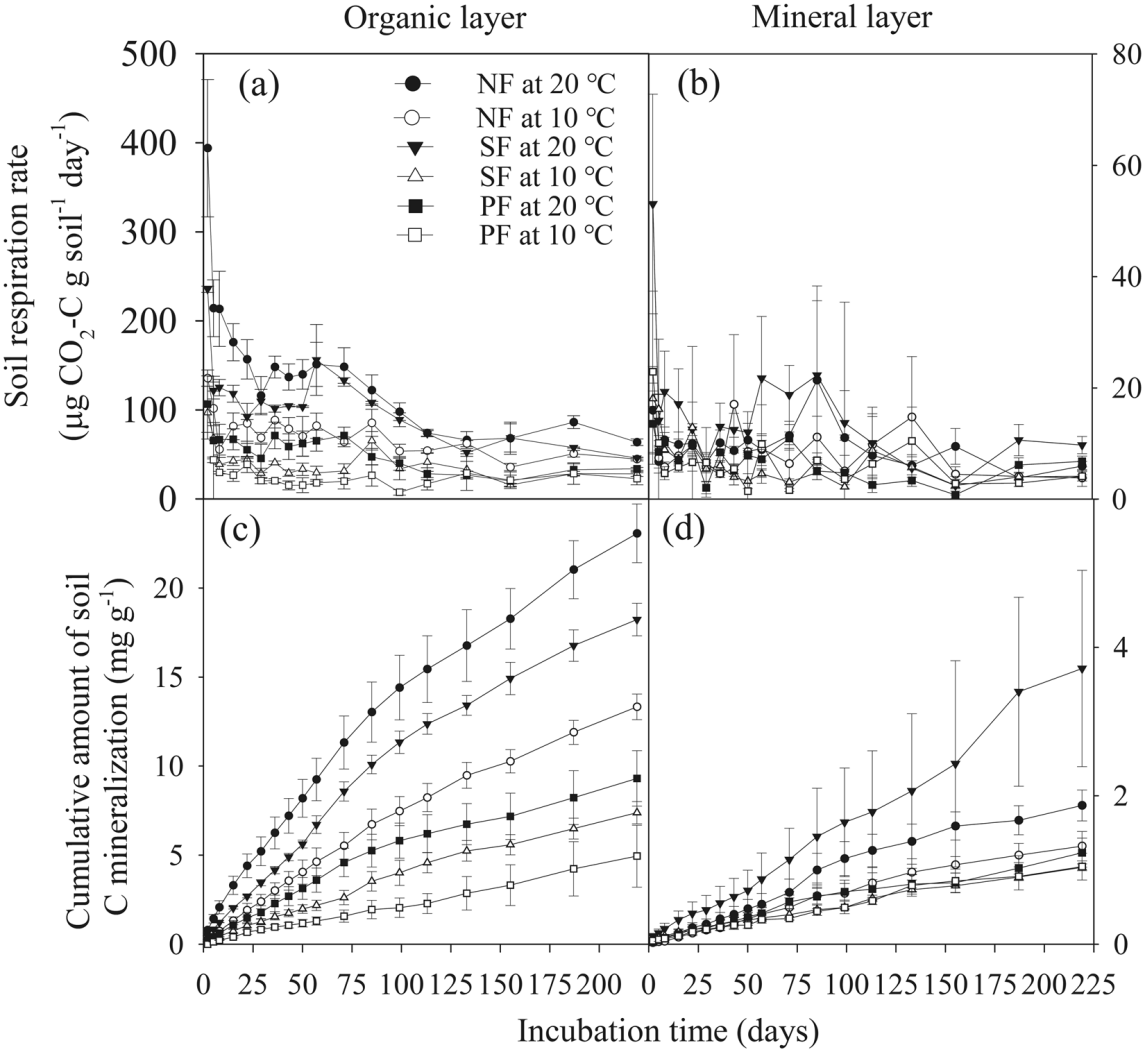
Effects of forest type conversion, soil layer and incubation temperature on soil C mineralization rates and cumulative C production. Values indicate means ± SE, n = 4. NF: natural forest, SF: secondary forest and PF: spruce plantation.

Forest type, incubation temperature, soil layer and time all had significant effects on soil C mineralization rate and cumulative C production (Fig. 3, Table 2). In the organic layer, both soil C mineralization rate and cumulative amount of C mineralization at 20 °C were higher than those at 10 °C on most of measurements (Fig. 3a,c). However, temperature often did not affect mineral soil C mineralization (Fig. 3b,d). Irrespective of incubation temperature, soil C mineralization rate was remarkably higher in the organic layer than in the mineral soil in each forest type (Fig. 3a–d, Table 2). In the organic layer, both soil C mineralization rate and cumulative C release followed a pattern of NF > SF > PF during the incubation period (Fig. 3a–d). The ANOVA results indicated that temperature effect on soil C mineralization was dependent on forest type and soil layer (Table 2).

**Table 2:** Results of four factors ANOVA showing the *F* and *P* values for responses of soil C mineralization rate and cumulative C production to incubation time (IT), forest type (FT), temperature (T) and soil layers (SL).

Factor	*d.f*.	Soil C mineralization rate		Cumulative C production	
		*F*	*P*	*F*	*P*
T	1	431.53	< 0.001	815.29	< 0.001
FT	2	200.22	< 0.001	377.45	< 0.001
SL	1	1883.49	< 0.001	3710.8	< 0.001
IT	17	25.2	< 0.001	182.92	< 0.001
T × IT	17	10.22	< 0.001	20.99	< 0.001
SL × IT	17	17.56	< 0.001	107.52	< 0.001
FT × IT	34	3.85	< 0.001	10.49	< 0.001
T × SL	1	303.87	< 0.001	558.55	< 0.001
T × FT	2	24.54	< 0.001	44.91	< 0.001
FT × SL	2	177.68	< 0.001	326.11	< 0.001
T × SL × IT	17	7.76	< 0.001	12.74	< 0.001
T × FT × IT	34	1.49	< 0.05	1.59	< 0.05
FT × SL × IT	34	4.38	< 0.001	8.28	< 0.001
T × FT × SL	2	13.62	< 0.001	22.31	< 0.001
T × FT × SL × IT	34	1.32	0.11	0.48	0.99

### Temperature sensitivity (*Q*_10_)

**Figure 4:**
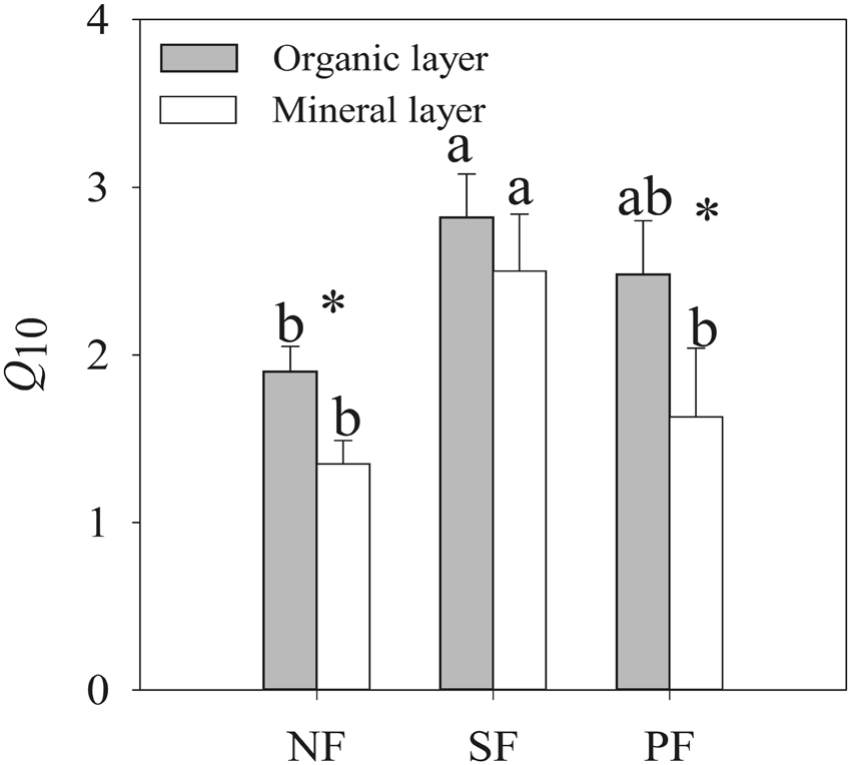
Effects of forest type conversion and soil layer on temperature coefficient *Q*_10_. Values indicate means ± SE, n = 4. Different letters within the same soil layer denote significant differences among forest types by one-way ANOVA. Asterisk indicates significant differences between the two soil layers by student *t*-test. NF: natural forest, SF: secondary forest and PF: spruce plantation.

Forest type and soil layer significantly affected *Q*_10_ values (Fig. 4, Tables 1 and 2). The *Q*_10_ varied from 1.35 to 2.82 across three forest types (Fig. 4). Irrespective of soil layer, *Q*_10_ was higher in the SF as compared to NF and PF. Meanwhile, *Q*_10_ values of organic layers were higher than those of mineral soils (Fig. 4). The statistical analysis showed that the interaction of forest type and soil layer was not significant on *Q*_10_ value (Table 1, *p* < 0.05).

### The correlations between soil substrates and C decomposition

SOC, N, P, bacteria and fungi were positively correlated with soil C mineralization rate (Table 3, all *p* < 0.001). However, pH, C:N and bacteria:fungi had a negative relationship with soil C mineralization rate (Table 3). Similarly, there were a marginal relationship between *Q*_10_ value and SOC, N, P and fungi (Table 3, all *p* < 0.1). Conversely, *Q*_10_ value decreased with increasing C:N and bacteria:fungi (Table 3, *p* < 0.1).

**Table 3:** Pearson correlations between soil properties and C decomposition rates and *Q*_10_ values.

Soil properties	Soil C mineralization rate		*Q*_10_	
	*r*	*p*	*r*	*p*
SOC	0.729	< 0.001	0.432	0.073
N	0.672	< 0.001	0.317	0.085
P	0.670	< 0.001	0.319	0.083
pH	−0.348	< 0.05	0.131	0.605
C:N	−0.450	< 0.001	−0.409	0.092
C:P	0.476	< 0.05	0.437	0.070
Bacteria	0.703	< 0.001	0.248	0.321
Fungi	0.721	< 0.001	0.405	0.096
Bacteria:Fungi	−0.292	0.084	−0.449	0.062

## Discussion

Forest land-use change could affect soil C mineralization directly and/or indirectly thought altering soil substrate conditions, including soil C quantity and quality, substrate availability and microbial properties^18–20^. A number of studies have reported that the conversion from natural forests to secondary forests and/or plantations decreased soil C pool, leading to lower soil C mineralization rates^18, 21, 22^. For instance, the secondary forest had higher C pools, microbial biomass and C mineralization rate as compared to larch plantations in Northeast China^18^. In this study, irrespective of incubation temperature, soil C mineralization rates in the organic layer generally followed a tendency of NF > SF > PF over the incubation period. This could be mainly attributed to the changes in soil substrate and microbial properties following the forest type conversion. SOC and N pools were decreased following the conversion from NF to PF. Additionally, both fungi and bacteria are two dominant microbial decomposer groups controlling soil C mineralization^23, 24^. Our results found that both soil fungi and bacteria PLFAs were markedly higher in the NF and SF than in the PF. This was also supported by statistical analysis because there were significant positive correlations between soil C mineralization rate and SOC, N and microbial PLFAs. Besides, it has been demonstrated that high-quality SOC is of great benefit to microbial carbon use efficiency^7^. Similar to previous studies^25, 26^, our results have also shown that soil C mineralization rate is negatively linked to C:N.

In boreal forests, there is an obvious organic layer accumulated in the upper forest floor due to slow decomposition. There are significant differences in soil biochemical properties between organic layer and mineral soil due to different rates of C input, accumulation, and turnover^13, 14^. Therefore, soil C decomposition rate could differ largely between two soil layers^27^. In this case, soil C mineralization rate was markedly greater in the organic layer compared to mineral soils in each forest type. This result was consistent with the observations from other boreal ecosystems^13, 27^. This is mainly because soil C pool and microbial PLFAs are extremely higher in the organic layer relative to mineral soil. It is widely accepted that SOC and microbial biomass directly regulate soil C mineralization in terrestrial ecosystems. Forest management practice profoundly and directly alters litter inputs which control substrate availability and quality for soil C decomposition^7, 20^. Previous studies have reported that fine roots mainly distributed in the organic layer in Tibetan forests^28^. Apparently, organic layer is much more vulnerable to forest land-use change as compared to mineral soils. Our results found that forest land-use change caused significant effects on soil biochemical properties, especially in the organic layer.

In recent years, the temperature sensitivity of soil C decomposition has gained much more attention^29, 30^. The temperature sensitivity of soil C decomposition, the factor by which soil C decomposition rate increases by 10 °C increases, is a key parameter to evaluate the feedback intensity between soil C efflux and climate change. Temperature coefficient *Q*_10_ is the most common measure to assess the temperature sensitivity of SOM decomposition in empirical studies. In the present case, forest land-use change may affect *Q*_10_ value via altering soil substrate availability and lability^20, 27^. In this study, *Q*_10_ values varied from 1.35 to 2.82 among three forest soils. A current synthesis has demonstrated that the *Q*_10_ values ranged from 1.10 to 5.18 across China’s forests^30^. Forest type conversion completely change dominant tree species and litter type, consequently affecting soil substrate and microbial properties, which are closely associated with *Q*_10_ value^7^. For example, the conversion from a primary forest dominated by *Quercus liaotungensis* to artificial plantations (*Larix principis-rupprechtii* and *Pinus tabulaeformis*) or secondary shrub forest significantly lowered *Q*_10_ value in northern China^31^. However, our results found that forest type conversion increased *Q*_10_ value. However, soil microbial biomass and C release rate were increased after conversion from native broadleaf forest to plantations in subtropical region^32^. Such differences imply that the effects of forest land-use change on soil C decomposition may vary with climatic zones.

On the other hand, some studies have reported that *Q*_10_ values increased with soil profile, reflecting that a decrease in substrate lability with soil depth^27, 33^. However, our results observed that *Q*_10_ value was greater in the organic layer as compared to mineral soil. This result was consistent with the findings observed in other boreal soils at high latitudes^13, 27^. Compared to mineral soils, there was a sharp reduction in soil C decomposition rate in the organic layers during the initial period of the incubation, implying that organic layer contained a small pool of very labile C pools, which was rapidly depleted over the early period of the incubation. Similar patterns have been observed in other boreal soils^13, 27^. Moreover, it was believed that the larger *Q*_10_ in the organic layer relative to mineral soil may be attributed to the extremely higher C availability, which may cause a decrease in the “cancelling effect”^13^. Finally, our study also showed that *Q*_10_ was positively associated with fungi PLFAs but negatively with bacteria:fungi ratios. Greater activation energy could be required for soil C mineralization when microbial activity is low, which may partially contribute to a higher *Q*_10_.

### Conclusions and implications

In summary, this study explored variations of soil C mineralization rate and its temperature sensitivity in the organic layer and mineral soil among three contrasting forests. Our results revealed that forest land-use change caused significant changes in substrate properties (e.g., C pools and PLFAs), especially in the organic layer. Both forest type and soil layer significantly influenced soil C mineralization rate and *Q*_10_ value. Taken together, the results demonstrated that soil C mineralization and its temperature sensitivity was a complex process that was susceptible to both direct and/or indirect controls derived from forest type conversion.

The findings in this study have the following important implications. On the one hand, forest management practice dramatically reduced soil C pools in both organic and mineral soils but significantly increase the *Q*_10_ of soil C decomposition. Thus, effective measures should be taken to manage the current primary forests to mitigate warming in this specific area. On the other hand, because soil C pool stored in the organic layer is very large in boreal forests and global warming is relatively pronounced in the surface layer. The higher *Q*_10_ value of the organic layer highlights its importance in boreal forests under a warming scenario. The differences in temperature response between the two layers should be considered when predicting soil C dynamic in boreal forests under a warmer world.

## Materials and Methods

### Study area

This study was conducted at the Long-term Research Station of Alpine Forest Ecosystems, which is located in the eastern Tibetan Plateau, China (102°53′–102°57′E, 31°14′–31°19′N). Mean temperature ranges from 2 to 4 °C and annual mean precipitation equals 850 mm. The soil was classified as dark brown forest soil with a 10–15 cm deep organic matter layer. Natural coniferous forest (NF), secondary birch forest (SF) and dragon spruce plantation (PF) are three dominant forest types due to local forest management practice. In July 2015, four 20 × 20 m plots were randomly established in each forest type. The basic conditions of three forests were recorded (Supplementary Table S1).

### Soil sampling

Soil samples of the organic layer and the upper mineral soil (10 cm) were collected in each plot. The organic layer was identified from the mineral soil via its color, texture and consistency^13^. Nine cores (5 cm diameter) were taken randomly from each plot and soil samples from same layer were mixed to get one composite sample. Each composite sample was passed through a sieve, and any visible living plant material was removed manually from the sieved soil. The sieved soil was kept in the refrigerator at 4 °C prior to the analysis of microbial properties. A sub-sample of each soil was air-dried and ground prior to chemical analysis.

### Sample analyses

Soil organic carbon (SOC) was measured using the dichromate oxidation sulfate ferrous titration method. Soil nitrogen (N) was analyzed following the Kjeldahl digestion procedure. Soil phosphorus (P) was determined using the phosphomolybdenum yellow colorimetry method. Soil pH was measured with a Calomel electrode at 1:5 soil-to-water ratio. The phospholipid fatty acids (PLFAs) were extracted and quantified using a modified method previously described by White^6^. Bacteria were identified by the following PLFAs: i15:0, a15:0, 16:0, 17:0, a17:0, 16:1w7c, 15:0, 16:1w5t, i17:0, 16:1w9c, 18:1w7c, 18:00, cy19:0, cy17:0, i16:0 and 20:5. Fungi was determined by the PLFAs 18:3, 18:2w6,9c, 18:1w9c and 20:1w9c.

### Soil C mineralization

Fresh soil samples (100 g) of the two layers were adjusted to 60% water holding capacity, which was considered optimal for microbial activity^34^. The soil samples were incubated in 1 L jars at two temperatures (10 °C and 20 °C). Empty jars without soils were used as controls. CO_2_ production was measured on 2, 5, 8, 15, 22, 29, 36, 43, 50, 57, 71, 85, 99, 113, 134, 155, 187 and 219 days after the incubation by using alkali absorption method. Soil samples were remoistened to keep moisture at each measuring time. The rate of soil C mineralization was calculated per unit mass in the unit time for average rate, and accumulative C production was the CO_2_ in the sum of unit time.

### Temperature sensitivity

The temperature sensitivity of soil C decomposition, the factor by which soil C decomposition rate increases by 10 °C increases, is a key ecological parameter in ecosystem carbon cycle models. Temperature coefficient *Q*_10_ is the most common measure to assess the temperature sensitivity of C decomposition in empirical studies. Therefore, *Q*_10_ was also applied in this study to compare temperature sensitivity of soil C decomposition among forest types using the method stated in Leifeld and Fuhrer^35^.

$${Q}_{10}={({R}_{20}/{R}_{10})}^{[10/W]}$$

Where *R*_20_ and *R*_10_ are the average C mineralization rates at 20 °C and 10 °C, respectively. *W* is the difference of incubation temperature.

### Statistical analysis

Four-way ANOVAs were employed to analyze the effects of forest type, soil layer, incubation temperature and time on soil C mineralization rates and accumulative C production. Two-way ANOVAs were used to test the effects of forest type and soil layer on measured soil variables and *Q*_10_ values. For same layer, one-way ANOVAs were used to identify significant differences in soil properties among forest types. For same forest type, student *t*-tests were used to compare the effect of the soil layer. The correlations between soil respiration rate, cumulative C production and *Q*_10_ and soil biochemical properties were analyzed by Pearson coefficient. The statistical tests were considered significant at the *p* < 0.05 level. All statistical tests were performed using IBM SPSS Statistics 20.0.

## Electronic supplementary material

Supplementary information
