# Supplementary material for: Temperature response of soil carbon decomposition depends strongly on forest management practice and soil layer on the eastern Tibetan Plateau
Source: Sci Rep. 2017 Jul 6;7:4777. doi: 10.1038/s41598-017-05141-2 (PMC5500495; doi:10.1038/s41598-017-05141-2)
Supplement: Supplementary file 1 — Supplementary information [file 41598_2017_5141_MOESM1_ESM.docx]

**Supplementary Table S1** Basic properties of three forest ecosystems on the eastern Tibetan Plateau. NF: natural forest, SF: secondary forest and PF: spruce plantation.

| Forest type | Soil type | Age (yr) | Dominant  species | Dominant  understory | Coverage | Organic soil  depth (cm) |
| --- | --- | --- | --- | --- | --- | --- |
|  |  |  |  |  |  |  |
| NF | Dark brown forest soil | >150 | *Abies*  *faxoniana* | *Rosa sweginzowii*, *Fargesia spathacea*, *Cystopteris montana*, *Carex spp* | 0.9 | 14.0±1.8 |
| SF | Dark brown forest soil | ～70 | *Betula albosinensis* | *Fargesia spathacea*, *Parasenecio forrestii*, *Thalictrum spp* | 0.8 | 10.4±2.0 |
| PF | Dark brown forest soil | ～60 | *Picea*  *asperata* | *Berberis diaphana*, *Deyeuxia scabrescens* | 0.8 | 9.3±2.2 |
|  |  |  |  |  |  |  |
